# Coursing the mottled mosaic: Generalist predators track pulses in availability of neonatal ungulates

**DOI:** 10.1002/ece3.10378

**Published:** 2023-07-26

**Authors:** Katey S. Huggler, Matthew M. Hayes, Patrick W. Burke, Mark Zornes, Daniel J. Thompson, Patrick Lionberger, Miguel Valdez, Kevin L. Monteith

**Affiliations:** ^1^ Haub School of Environment and Natural Resources Wyoming Cooperative Fish and Wildlife Research Unit Department of Zoology and Physiology University of Wyoming Laramie Wyoming USA; ^2^ Wyoming Game and Fish Department Green River Region Green River Wyoming USA; ^3^ Wyoming Game and Fish Department Large Carnivore Section Lander Wyoming USA; ^4^ Bureau of Land Management Rock Springs Field Office Rock Springs Wyoming USA

**Keywords:** *birth pulse*, Canis latrans, *coyote*, Odocoileus hemionus, *optimal foraging*, *resource tracking*

## Abstract

The density and distribution of resources shape animal movement and behavior and have direct implications for population dynamics. Resource availability often is “pulsed” in space and time, and individuals should cue in on resource pulses when the energetic gain of doing so exceeds that of stable resources. Birth pulses of prey represent a profitable but ephemeral resource and should thereby result in shifting functional responses by predators. We evaluated movements and resource selection of coyotes (*Canis latrans*) across a gradient of reproductive stages ranging from late gestation to peak lactation of female mule deer (*Odocoileus hemionus*) in southwest Wyoming, USA, to test whether coyotes exhibited shifts in selection and movement behavior relative to the availability and vulnerability of neonatal mule deer. We expected coyotes to track pulses in availability of neonatal mule deer, and such behavior would be represented by shifts in resource selection and search behavior of coyotes that would be strongest during peak parturition of mule deer. Coyotes selected areas of high relative probability of use by female mule deer and did so most strongly during peak parturition. Furthermore, searching behavior of coyotes intensified during pulses of availability of deer neonates. Our findings support the notion that coyotes exploit pulses of neonatal deer, presumably as an attempt to capitalize on a vulnerable, energy‐rich resource. Our work quantifies the behavioral mechanisms by which coyotes consume ungulate neonates and provides one of the first examples of a mammalian predator–prey system centered on a pulsed resource.

## Graphical abstract

Birth pulses of prey represent a profitable, ephemeral resource that should be sought after by small carnivores. Coyotes are a common predator of neonatal ungulates, yet the mechanisms underpinning such predation events are unknown. Our analyses indicate that coyotes exploit pulses of neonatal deer by shifting selection and altering movement strategies to capitalize on an energy‐rich resource.

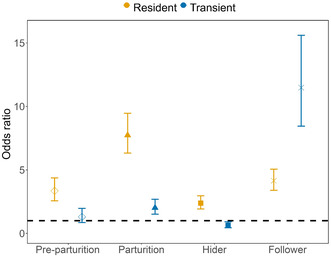

## 1 INTRODUCTION

Spatial and temporal variation in resources is the foundation in which animal movement and space use operate. Multiple external factors, such as environmental conditions and risk of predation, can limit access to resources (Brown, 1999; Pyke et al., 1977). The ability to acquire sufficient resources has direct implications for life‐history functions (e.g., reproduction and mating), and vital rates (e.g., fecundity, litter size, and neonate survival) of many taxa (Fuller & Sievert, 2001). Consequently, variation in resource availability should encourage animals to move within home ranges in a manner that satisfies access to adequate food resources while balancing other risks. Resources not only vary across space but also can vary temporally (Armstrong et al., 2016; Yang et al., 2008). Temporal variation in food resources should promote plastic movement tactics in which individuals attempt to capitalize on windows of resource availability (Aikens et al., 2017; Armstrong et al., 2016; Deacy et al., 2016; Schindler et al., 2013), especially if resources that are temporally variable are sufficiently more profitable than stable resources (Aikens et al., 2017; Armstrong et al., 2016; Schindler et al., 2013).

Optimal foraging theory provides the context for understanding animal space use and behavior; and why resource pulses should be sought after by consumers. Animals that forage optimally should switch resources when the benefits of capturing that resource outweigh the costs (Charnov, 1976; Emlen, 1966; Stephens & Krebs, 1986; Walker et al., 2022). Increased energetic expenditure associated with elevated search effort, movement costs, or handling time are costs that may be incurred by tracking resource pulses. Moreover, tracking resource pulses may increase the risk of mortality to a consumer if pulses are spatially or temporally unpredictable or when exploiting such resource pulses requires entering territories of competitors. Nevertheless, tracking resource pulses provides energetic gain that can exceed that of total resource abundance alone. Consumers may increase resource gain up to 1000 percent by exploiting resource pulses while simultaneously reducing energy expenditure necessary to acquire that resource (Aikens et al., 2017; Armstrong et al., 2016; Ruff et al., 2011).

Pulses in resource availability may be especially advantageous to carnivores whose prey are mobile, difficult to locate, and difficult to capture once located (Stephens & Krebs, 1986; Svoboda et al., 2019). Prey breadth of predators often is limited by body size of prey that the predator can capture—prey that are too large are difficult and dangerous to capture. In particular, small predators (<12 kg) are typically limited to prey that are no more than 45 percent of their body weight (Carbone et al., 1999; Petroelje et al., 2013); thus, prey exceeding the limitations of body size of a predator likely are not energetically advantageous (Petroelje et al., 2013). Furthermore, social cohesion of predators (e.g., residents versus transients) may play an important role in determining the size of prey that predators are able to capture (Gifford et al., 2017). For instance, group size of coyotes (*Canis latrans*) was largest during winter, which coincided with diet shifts from rodents to ungulates (Gese et al., 1988). Conversely, during summer, group size of coyotes was small which coincides with the availability of vulnerable, neonatal prey (Gese et al., 1988). Birth pulses of prey provide a window of opportunity that should be sought after by small predators regardless of social status: prey are vulnerable and clumped, so search and handling time are minimized, and profitability is maximized.

Ungulates exhibit marked birth pulses wherein neonates are born within a short period of time, offering a pulse of vulnerable prey (Bastille‐Rousseau et al., 2011; DeMars et al., 2013; Duquette et al., 2015; Monteith et al., 2014), which should be a profitable resource for predators. Coyotes are common predators of neonatal ungulates (Kilgo et al., 2012; Linnell et al., 1995; Robinson et al., 2014), but it is unclear whether coyotes opportunistically encounter neonate prey, or actively seek neonates during birth pulses. We evaluated whether coyotes track pulses in availability of neonatal mule deer (*Odocoileus hemionus*) in a high‐desert system in the Intermountain West. We tested the hypothesis that movement and space use by coyotes would vary temporally with availability of neonatal mule deer and that territorial strategies of coyotes would influence space use and movement in relation to availability of neonatal mule deer. We predicted that coyotes (residents and transients) would consistently select areas of high relative probability of use by mule deer, but selection would be strongest during peak parturition when neonates are most abundant and vulnerable, and selection by coyotes would remain high immediately postparturition when neonates are still vulnerable to predation. Furthermore, we expected area‐restricted search behaviors by coyotes to be associated positively with relative probability of use by mule deer, and that search behavior would be most intense during peak parturition.

## 2 STUDY AREA

Our study occurred in the Greater Little Mountain Ecosystem (6400 km^2^; 41°4′26.22″ N 109°3′1.80″ W) located in southwest Wyoming, USA, during late spring and summers of 2018 and 2019. Our study area was a high‐desert system with low elevation (~1800 m) valleys of mixed sage‐grassland (*Artemisia* spp., blue‐bunch wheatgrass [*Pseudoroegneria spicata*], and cheatgrass [*Bromus tectorum*]) that transitioned into patchworks of pinyon‐juniper (*Juniperus* spp.) at intermediate elevations (~2400 m) and pockets of quaking aspen (*Populus tremuloides*) and subalpine fir (*Abies lasiocarpa*) at high elevations (~2700 m). Climate was characterized by long, and often severe winters and short, dry summers. Snowfall during winter 2017–2018 was less (92 percent of median snowpack) than that of 2018–2019 (103 percent of median snowpack; NRCS Snowpack Data). Average summer precipitation from May to July in 2018 was 2.7 and 3.6 cm in 2019. May precipitation in 2018 was well above the average from 1981 to 2010; however, precipitation plummeted to 45 percent of average by July 2018, producing drought‐like conditions across the study area (SNOTEL Data, Green River Station).

Coyotes, mountain lions (*Puma concolor*), and bobcats (*Lynx rufus*) were the most common predators in the study area, and black bears (*Ursus americanus*) occurred at relatively low densities. In our study area, coyotes were often found at kill sites made by mountain lions; however, coyotes did not alter fine‐scale movement behavior when in close proximity to mountain lions, indicating that coyote behavior likely was not constrained by the presence of other predators (Brunet et al., 2022).

Several ungulate species were present in the study area, including elk (*Cervus elaphus*), mule deer, pronghorn (*Antilocapra americana*), feral horses (*Equus ferus*), and Shiras moose (*Alces alces shirasi*); however, the density of feral horses and Shiras moose was relatively low (Huggler et al., 2022). Small mammals in the study area that were common diet items for coyotes include cottontail rabbits (*Sylvilagus* spp.), jackrabbits (*Lepus* spp.), voles (*Microtis* spp.), and white‐tailed prairie dogs (*Cynomys leucurus*).

## 3 MATERIALS AND METHODS

We collected GPS data from radio‐collared coyotes and female mule deer (hereafter, referred to as mule deer) from April to September of 2018 and 2019. During April 2017–January 2018, we captured coyotes via foothold traps (Minnesota Brand 550, 4‐coil with rubber jaws) and fitted them with a GPS radio collar (Advanced Telemetry Systems, Inc. Isanti, Minnesota, USA) programmed to take a location every hour. Additionally, during April 2017–2019, we captured coyotes and mule deer with a handheld net gun fired from a helicopter (Barrett et al., 1982; Jacques et al., 2009; Webb et al., 2008). Each animal was transported to a central processing location where we fit them with a GPS radio collar (Advanced Telemetry Systems, Inc. Isanti, Minnesota, USA or Vectronics Aerospace, Berlin, Germany) programmed to collect locations at 1‐h intervals. Additionally, we determined sex and estimated age of coyotes via tooth wear (Bowen, 1982; Gier, 1957).

We assessed pregnancy status and number of fetuses of mule deer during each April capture using ultrasonography with transabdominal scanning using a 3‐MHz transducer (Ibex Pro Portable Imaging Device, E.I. Medical Imaging, Loveland, CO, USA; Monteith et al., 2014; Stephenson et al., 1995). If pregnant, we fit them with a Vaginal Implant Transmitter (VIT, Advanced Telemetry Systems, Inc., Isanti, Minnesota, USA or Vectronics Aerospace Berlin, Germany) to assist in identifying precise timing of birth events. VITs were equipped with a UHF communication system in which the VIT communicated with the dam's collar and sent an email notification upon expulsion of a VIT. Upon receipt of an expulsion email, we located the VIT via radio telemetry and identified if the location of a VIT was a birth site. Birth sites were confirmed based on evidence of remaining afterbirth, matted grass or dirt, and the presence of neonate(s). Animal capture and handling methods were in accordance with guidelines from the American Society of Mammalogists (Sikes, 2016) and approved by the University of Wyoming Institutional Animal Care and Use Committee (20170322KM00267; 20170404KM00270).

We subset GPS data of mule deer into four periods that corresponded with major changes in reproductive status and mobility of offspring: Pre‐Parturition, Parturition, Hider, and Follower. We defined reproductive periods for individual mule deer from confirmed birth events, and each reproductive period consisted of 37 days of GPS data. We chose 37 days as the time window because that was the time window in days wherein 95% of mule deer births occurred in our study area. Furthermore, the subset of 37 days for reproductive periods ensured that our analyses of the relative probability of use by mule deer and coyotes aligned temporally. We defined the Parturition period for each mule deer as the 37‐day window after their parturition date, and we defined the Pre‐Parturition period as the 37 days before their parturition date. To avoid issues with spatiotemporal autocorrelation, we thinned hourly GPS locations of mule deer to 5‐h fix rates.

We elected to include a Hider phase to represent the period in which most neonates still remain as hiders and are still relatively vulnerable to predation if found (Bowyer et al., 1998; Carl & Robbins, 1988; Lent, 1974). Therefore, the Hider period was defined as the 37 days after the last day of the Parturition period. Additionally, we defined the next 37‐day period as a Follower phase to represent the period in which neonates have gained mobility and are less vulnerable to predation by coyotes.

We subset GPS data of coyotes to align with the reproductive periods of mule deer at the population level. Using parturition dates collected from confirmed birth events, we defined Parturition as the date range wherein 95 percent of births occurred in 2018 and 2019. We then calculated the time difference (in days) between the beginning date of the Parturition period and the end date of the Parturition period for both years, so that the time window remained consistent across years. Consequently, each reproductive period was 37 days in length. We used the calculated time difference of 37 days to subset the remaining reproductive periods. The Pre‐Parturition period was defined as the 37 days before the first day of Parturition, whereas the Hider period was defined as the 37 days after the last day of Parturition, and the Follower phase was defined as the 37 days after the last day of the Hider phase.

### 3.1 Predicted use by mule deer

We modeled the relative probability of use by mule deer at the 2nd‐order (i.e., landscape) scale (Johnson, 1980; Manly et al., 2007) for each of the four reproductive periods. We created 95% home ranges for each mule deer using GPS data using autocorrelated kernel density estimation procedures in the “ctmm” package version 0.6.1 (aKDE; Calabrese et al., 2016; Fleming & Calabrese, 2017; Fleming et al., 2015; Noonan et al., 2019). We determined the number of available locations necessary to represent variation in the landscape using methods described by Long et al. (2014). We determined that 2000 total available locations per reproductive period were sufficient for quantifying habitat availability in our study area. GPS locations of female deer represented “used” locations. We cast 2000 random locations within each home range to index availability.

We modeled the relative probability of use by mule deer using habitat and topographic variables expected to influence the relative probability of selection by mule deer, including distance to primary roads, distance to secondary roads, slope (degrees), cosine of aspect (northness), sine of aspect (eastness), elevation, and percent cover estimates of shrubs, forest, perennials, and litter (Jones et al., 2018). We modeled all variables at 30 m resolution. We modeled the relative probability of use by mule deer for each of the four reproductive periods using a machine learning approach, random forest (RF). Random forest is a nonparametric machine learning approach that does not rely on normality, can handle complex interactions and variables that are correlated, and generally outperforms traditional models when prediction is the primary aim (Breiman, 2001; Cutler et al., 2007; Murphy et al., 2010; Shoemaker et al., 2018). Furthermore, RF models are based on decision trees that use a voting system to determine variables that best classify categories (0 = available, 1 = used, in our instance).

We used 90 percent of the sampled GPS data as the training dataset for the RF model and withheld the remaining 10 percent as our test dataset. RF models can be tuned by identifying the number of trees to build, and the number of variables to test when splitting decision trees; also known as the “mtry” parameter. Tuning RF models is a method to optimize the predictive accuracy of a model to a particular dataset. Using the caret package (version 6.0–84) in R version 3.5.2, we ran 10 iterations of a RF model on our training dataset and allowed for up to four random variables to be used to split decision trees at any one time. We used predictive accuracy (i.e., the proportion of correctly predicted values) as the metric to optimize within the training model. We used the final tuned RF model in which predictive accuracy was maximized to predict back to the remaining 10 percent of our withheld test data.

A probability of 0.5 is the default cutoff for classifying whether a point is used (1) or available (0). Such probability, however, is somewhat arbitrary and may not be appropriate to minimize classification error (Hanley & McNeil, 1982). Consequently, we constructed a receiver operating characteristic (ROC) curve using the pROC package (version 1.13.0) in R version 3.5.2 (R Core Team, 2018) to identify the probability cutoff value in which the false‐positive rate is minimized and true‐positive rate is maximized. Finally, we used the optimal “mtry” value from the tuned model and the best probability cutoff value for the final prediction that included all our data. We used the final model to predict the relative probability of use by mule deer across the landscape for each of the four reproductive periods.

### 3.2 Social status of coyotes

We determined social status (resident or transient) of coyotes using ≥3 months of space use by coyotes (Hinton et al., 2015; Webster et al., 2022) and a rarefaction curve for each animal that was created by estimating monthly home ranges (Dellinger et al., 2013; Webster et al., 2022). We classified “residents” in a particular year (from Pre‐Parturition to the Follower phase) as coyotes that showed stable space use for ≥3 months. Conversely, “transient” coyotes were defined as coyotes who exhibited unstable space use over time. We estimated 95% monthly home ranges and transient ranges using autocorrelated kernel density estimation (aKDE; Calabrese et al., 2016; Fleming & Calabrese, 2017; Fleming et al., 2015; Noonan et al., 2019). We were unable to determine social status of coyotes if they did not have at least 3 months of GPS data available; therefore, if we were unable to determine social status of a coyote for a particular year, they were excluded from analysis. Transient coyotes do not maintain territories; therefore, we refer to space use patterns by transients as biding areas hereafter (Webster et al., 2022).

### 3.3 Resource selection by coyotes

We evaluated resource selection by coyotes at the 3rd‐order (i.e., within home ranges or biding areas) scale (Johnson, 1980; Manly et al., 2007) during the four reproductive periods of mule deer. We first created 95% autocorrelated kernel density home ranges or biding areas (aKDE; Calabrese et al., 2016; Fleming & Calabrese, 2017; Fleming et al., 2015; Noonan et al., 2019) for each coyote‐year. Identical to methods described for mule deer, we sampled 2000 available points within each coyote home range or biding area using the sp package (version 1.3‐1) in Program R version 3.5.2 (R Core Team, 2018). We considered several covariates in our models, including distance to primary roads, distance to secondary roads, slope (degrees), cosine of aspect, sine of aspect, elevation, and percent cover estimates of shrubs, forest, perennials, and litter (Jones et al., 2018), and the corresponding layer of the relative probability of use by mule deer for that reproductive period. We tested for correlations among all covariates using a correlation matrix. Any variables that were correlated (|*r*| > .6) were not included in the same model. We used package lme4 (version 1.1‐18‐1) in Program R version 3.5.2 (R Core Team, 2018) to test binomial generalized linear mixed‐effects models using a maximum likelihood estimator. We classified used (1) and available (0) points as the response and the topographic and relative probability of use by mule deer as the fixed effects, with coyote id‐year as a random intercept to account for variation among individual coyotes and to reduce spatial autocorrelation (Bolker et al., 2009; Dormann et al., 2007; Gillies et al., 2006).

We developed a global model for each reproductive period that included topographic variables that were expected to influence selection by coyotes. No covariates were collinear (|*r*| > .6); therefore, we elected to include all covariates in our global model. For each reproductive period, we developed an independent model set that included: (1) a “global” model, (2) a “deer” model (including the global model with the addition of the corresponding (i.e., same temporal window) of deer use), and (3) a “deer × social status” model (including the interaction between deer use and social status (i.e., resident or transient) of coyotes; Table 1). Resident coyotes represented the reference category for our analyses. We used Akaike's information criterion adjusted for small sample size (AIC_*c*_) to determine whether including the respective deer layer and/or social status within each reproductive period improved model fit. We considered a covariate to have improved model fit if AIC_*c*_ decreased by >2 when it was included (Burnham & Anderson, 2004). We standardized all covariates before parsing data into reproductive periods by subtracting the mean and dividing by the standard deviation to allow for comparisons of the influence of variables on selection by coyotes. We deemed a covariate to effect selection by coyotes if 95 percent confidence intervals did not overlap zero. Finally, we interpreted the relationship between covariates in the model, and probability of selection by coyotes within each reproductive period using odds ratios (Neumann et al., 2012).

**TABLE 1:** Model results for resource selection of coyotes during Pre‐Parturition, Parturition, Hider, and Follower phases.

Reproductive period	Model	K	AIC*c*	ΔAIC*c*	ω*i*
Pre‐parturition	GLOBAL + DEER*STATUS	15	45933.35	0.0	0.99
	GLOBAL + DEER	13	45949.35	16.0	0
	GLOBAL	12	46004.68	71.33	0
Parturition	GLOBAL + DEER*STATUS	15	88150.09	0.0	1
	GLOBAL + DEER	13	88231.21	81.13	0
	GLOBAL	12	88581.79	431.70	0
Hider	GLOBAL + DEER*STATUS	15	84052.55	0.0	1
	GLOBAL + DEER	13	84101.34	48.78	0
	GLOBAL	12	84124.96	72.41	0
Follower	GLOBAL + DEER*STATUS	15	81244.28	0.0	1
	GLOBAL + DEER	13	81283.34	39.06	0
	GLOBAL	12	81734.34	490.05	0

*Note*: Terms in the global model included distance to primary roads, distance to secondary roads, topographic position index, slope, percent litter, percent shrub, percent perennial, and percent forest. The GLOBAL + DEER model included the global model plus the corresponding layer of deer, and the GLOBAL + DEER*STATUS included the global model plus the interaction between the corresponding deer layer and coyote social status. K = number of parameters in the model, ΔAIC_*c*_ = the difference between AIC_*c*_ of the top model and each additional model.

### 3.4 Search behavior by coyotes

We quantified search behavior of coyotes using the intensity of use metric (Almeida et al., 2010; Walker et al., 2022). Intensity of use is directly proportional to the time spent per unit area and will increase when coyotes exhibit complex paths or move slowly through an area (Almeida et al., 2010; Walker et al., 2022). Consequently, intensity of use represents the activity of coyotes within their home ranges and allowed us to test the prediction that coyotes exhibit search behavior during mule deer parturition. For each coyote‐year combination, we used a moving window approach to create a movement trajectory for every 12 h using the amt package (version 0.1.6) in Program R version 3.5.2 (R Core Team, 2018). We chose 12 locations as the subset for a trajectory because it allowed the path length to be long enough to preserve turning angles from the original path. From each trajectory, we calculated the intensity of use using the amt package in Program R version 3.5.2 (R Core Team, 2018). To relate the intensity of use values back to the relative probability of use by mule deer, we calculated the mean extracted value of the relative probability of use by mule deer for each GPS location in the trajectory and assigned the average value along with the reproductive period of that trajectory. The resulting dataset included one intensity of use value for every 12 points along with an average value of the relative probability of use for each reproductive period. To ensure that our interpretation of the relationship between intensity of use and relative probability of use by deer was not an artifact of differing movement strategies (i.e., resting vs. moving) throughout the diel period, we calculated movement metrics using only crepuscular locations to reflect the time wherein coyotes are most likely to be foraging (Andelt & Andelt, 1981; Youngmann et al., 2022).

We modeled the intensity of use in a linear mixed‐effects model framework with the nlme package (version 3.1‐137) in Program R version 3.5.2 (R Core Team, 2018). We modeled each reproductive period (i.e., Pre‐Parturition, Parturition, Hider, or Follower) separately, resulting in four models. Our models included the fixed effect of the interaction between relative probability of use by mule deer and coyote social status and a random intercept of coyote id‐year to account for autocorrelation and repeated measures among individuals (Bolker et al., 2009; Dormann et al., 2007; Gillies et al., 2006). We standardized the relative probability of use by mule deer before parsing data into reproductive periods by subtracting the mean and dividing by the standard deviation to allow for comparisons of the influence of the relative probability of use by mule deer on movement behavior of coyotes. We assessed the effects of the relative probability of use by mule deer by assessing whether 95 percent confidence intervals overlapped zero.

## 4 RESULTS

Most births (i.e., 95%) occurred between 20 May and 26 June in 2018 and 2019; each reproductive period thereafter for the resource selection and movement behavior by coyotes was subset around Parturition for each year‐Pre‐Parturition occurred from 13 April to 19 May, the Hider phase occurred from 27 June to 2 August, and the Follower phase occurred from 3 August to 8 September. Our analyses included a total of 56 mule deer and 27 coyotes. Overall, our analyses incorporated anywhere from 10,349 to 31,101 GPS locations of coyotes and 27,027 to 46,130 GPS locations of mule deer, depending on reproductive period (Appendix A: Table A1).

### 4.1 Resource selection by coyotes

**FIGURE 1:**
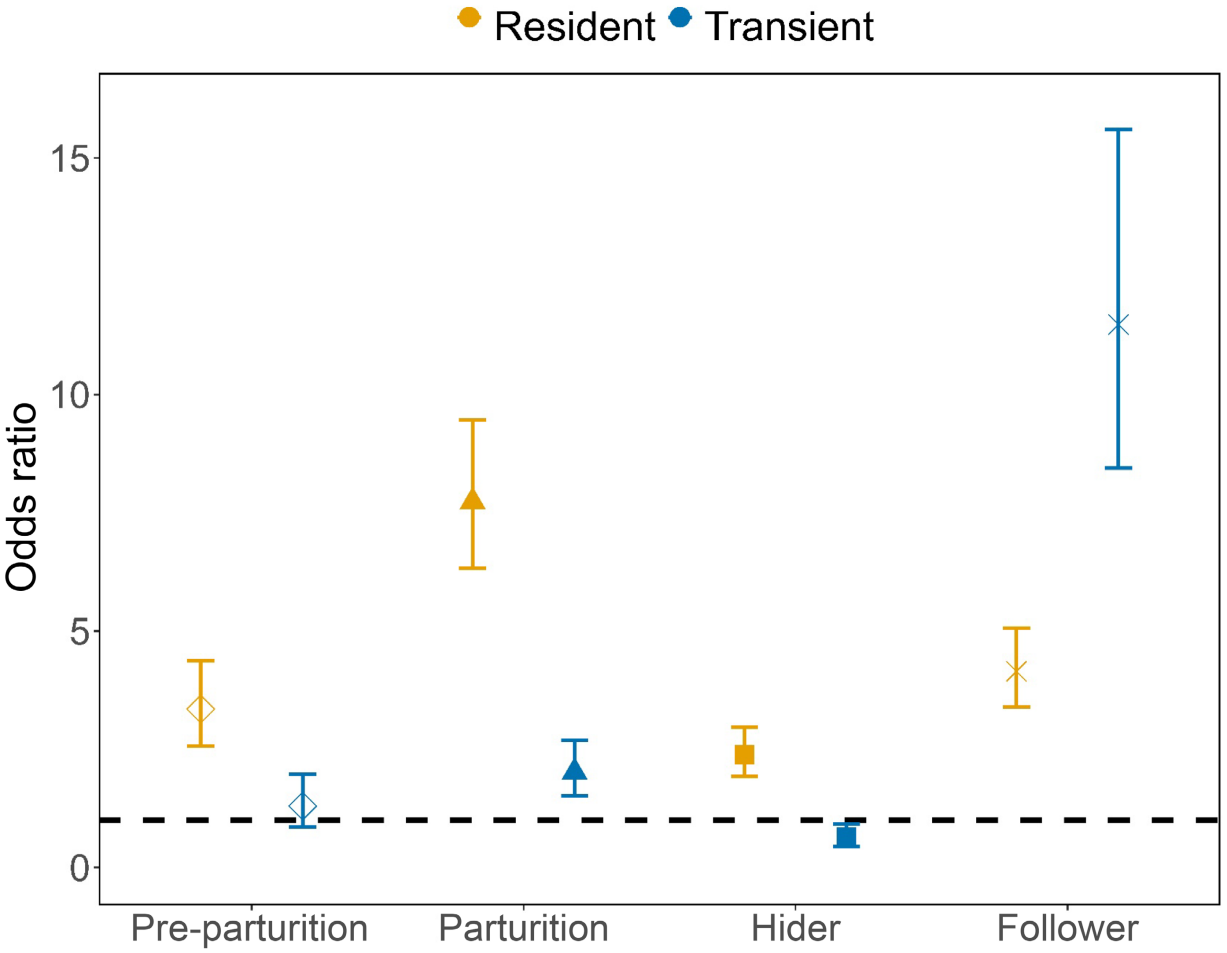
Odds ratio (±95% confidence intervals) of selection by coyotes to the relative probability of use by mule deer from Pre‐Parturition to the Follower phase.

Including the interaction between relative probability of use by mule deer and social status improved model fit (i.e., ΔAIC_*c*_ > 2) in all model sets (Table 1). The affinity of coyotes for areas of high relative probability of use by mule deer varied across reproductive periods. Resident coyotes selected areas likely to be used by mule deer more strongly than transients across all reproductive periods except for the Follower phase (Figure 1). Resident coyotes selected areas more likely to be used by mule deer across all reproductive periods, but selection was strongest during mule deer Parturition. During Parturition, an area used by a mule deer was 7.7 times more likely to be selected by a resident coyote than an area that was not used by a mule deer whereas odds of selection for areas used by mule deer was 3.3‐fold during the Pre‐Parturition phase and 4.1‐fold during the Follower phase (Figure 1). Selection by residents for areas used by mule deer was weakest during the Hider phase (2.3‐fold; Figure 1). Transient coyotes selected for areas more likely to be used by mule deer most strongly during the Follower phase, followed by the Parturition phase. Transients, however, were indifferent to areas most likely to be used by mule deer during Pre‐Parturition and avoided mule deer during the Hider phase (Figure 1).

Based on standardized selection coefficients, coyotes generally were indifferent to aspect across all reproductive phases. Coyotes selected low elevations during Pre‐Parturition (β = −0.362) and Parturition (β = −0.069) and selected high elevations during the Hider (β = 0.127) and Follower phases (β = 0.317). Coyotes selected areas two times farther from primary roads during Parturition (β = 0.047) compared with the Follower phase (β = 0.104) and were indifferent to primary roads during the Pre‐Parturition and Hider phases. Similarly, coyotes selected areas farther from secondary roads during Parturition (β = 0.032) and selected areas closer to secondary roads nearly two times stronger during the Hider phase (β = −0.078) compared with the Follower (β = −0.043) phase. Across all reproductive periods, coyotes avoided steep slopes with the strongest avoidance occurring during the Pre‐Parturition phase. Coyotes strongly selected perennial cover during all reproductive periods but did so most strongly during the Hider phase (β = 0.494). Selection of shrub cover increased sixfold from the Parturition (β = 0.044) to the Follower phase (β = 0.278). Coyotes avoided forest cover from Pre‐Parturition to the Hider phase, but selected forest cover during the Follower phase. During the Parturition phase, coyotes selected litter cover, and avoided litter from the Hider to the Follower phase (Table 2).

**TABLE 2:** Effects of landscape variables, relative probability of use by deer, and social status rank on within home range selection by coyotes.

Parameter	Pre‐parturition			Parturition			Hider			Follower		
	EST	95% CI		EST	95% CI		EST	95% CI		EST	95% CI	
		Lower	Upper		Lower	Upper		Lower	Upper		Lower	Upper
COSASPECT												
SINASPECT												
DEM	−0.362	−0.404	−0.32	−0.069	−0.100	−0.038	0.127	0.092	0.162	0.317	0.283	0.351
DIST2PRIM				0.047	0.018	0.076				0.104	0.074	0.135
DIST2SEC				0.032	0.015	0.049	−0.078	−0.098	−0.057	−0.043	−0.062	−0.024
SLOPE	−0.174	−0.206	−0.144	−0.148	−0.168	−0.128	−0.136	−0.156	−0.115	−0.099	−0.120	−0.079
SHRUB				0.044	0.024	0.064	0.172	0.152	0.192	0.278	0.257	0.298
TREE	−0.104	−0.137	−0.071	−0.122	−0.142	−0.101	−0.094	−0.116	−0.071	0.053	0.034	0.073
LITTER				0.092	0.073	0.111	−0.075	−0.095	−0.054	−0.046	−0.066	−0.025
PERENN	0.398	0.364	0.431	0.409	0.385	0.433	0.494	0.468	0.519	0.474	0.447	0.500
DEER	0.143	0.111	0.174	0.248	0.224	0.272	0.095	0.071	0.119	0.171	0.147	0.195
STATUS:TRANSIENT												
DEER*STATUS	−0.111	−0.161	−0.062	−0.163	−0.198	−0.128	−0.144	−0.183	−0.105	0.122	0.085	0.159

*Note*: The coefficients are expressed as standardized coefficients and 95% confidence intervals. Only parameters that were significant (*p* < .05) are presented. Parameters include cosine aspect (COSASPECT), sine aspect (SINASPECT), elevation (DEM), distance to primary roads (DIST2PRIM), distance to secondary roads (DIST2SEC), slope (SLOPE), percent shrub cover (SHRUB), percent forest cover (TREE), percent litter cover (LITTER), percent perennial cover (PERENN) as well as relative probability of use by mule deer (DEER), social status of coyotes (STATUS; Resident as the reference category), and the interaction between relative probability of use by mule deer and coyote social status (DEER*STATUS).

### 4.2 Search behavior by coyotes

**FIGURE 2:**
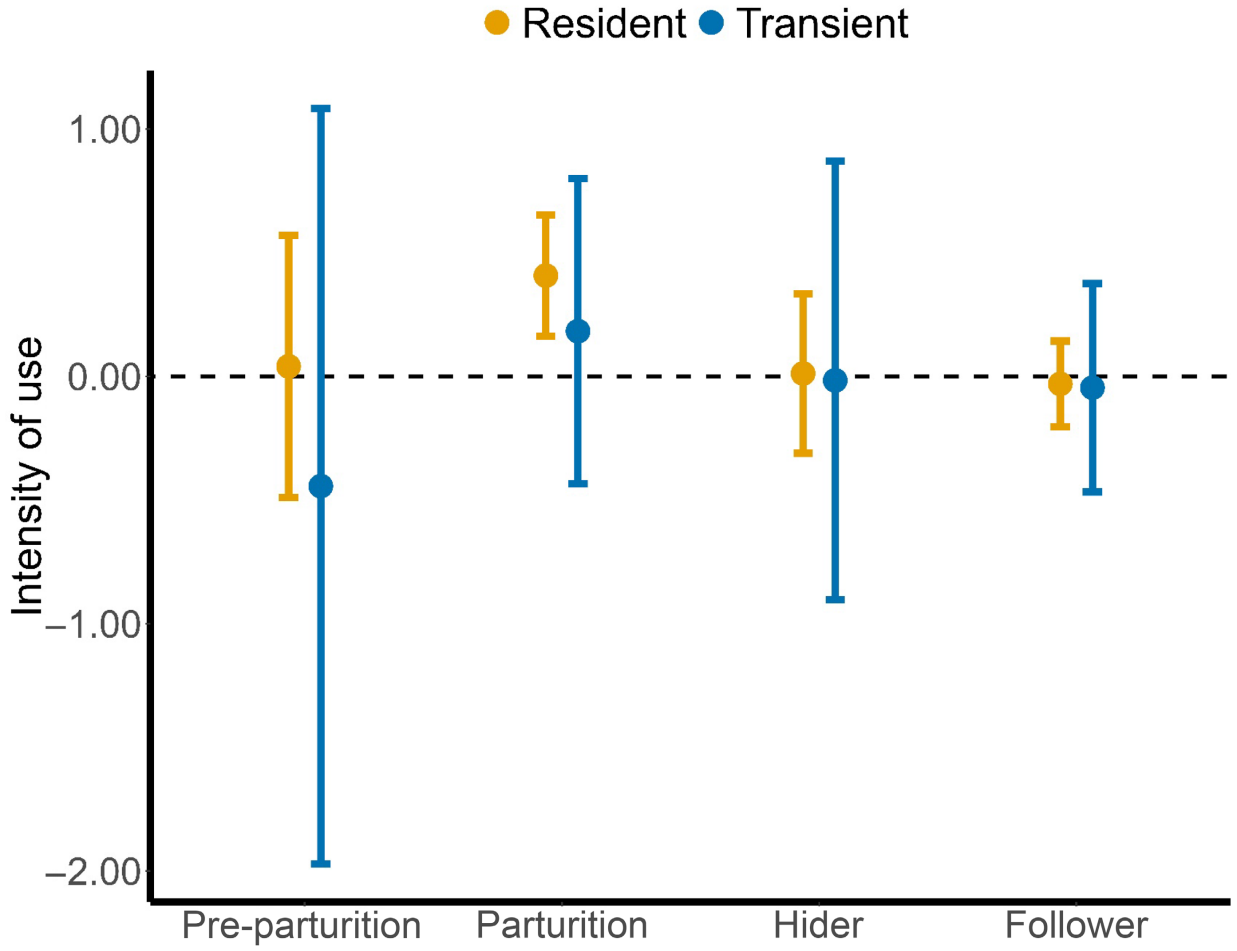
Standardized parameter estimates ±95% CI of the relationship between intensity of use and relative probability of use by mule deer across reproductive periods.

Resident coyotes exhibited stronger intensity of use in areas more likely to be used by mule deer during Parturition only. There was no relationship between intensity of use by resident coyotes and relative probability of use by mule deer during any other reproductive period. Relative probability of use by mule deer was unimportant in determining the intensity of use by transient coyotes during all reproductive periods (Figure 2).

## 5 DISCUSSION

We evaluated whether a generalist predator shifted patterns of resource use and fine‐scale movement to capitalize on a pulsed and highly vulnerable resource. Our findings were consistent with notions underlying optimal foraging theory in that coyotes seemingly searched in and increased selection for areas that were most likely to be rich in availability of neonates (Figures 1 and 2). The pervasive shifts in resource selection and search behavior by coyotes toward areas where neonates were located, which also aligned with the timing of when neonates were most abundant, indicate that coyotes attempted to increase consumption of neonates when availability of neonates peaked.

Consistent with our hypothesis, resident coyotes selected areas with high relative probability of use by mule deer during all reproductive periods, but selection was strongest during peak parturition. Residents were 7.7 times more likely to use an area also selected by mule deer than an area that was not used by a mule deer during Parturition (Figure 1). Contrary to our expectations, selection by resident coyotes toward mule deer waned after the birth pulse during the Hider phase, when neonates were presumably still vulnerable to predation (Bowyer et al., 1998). Reduced selection for mule deer by residents during the Hider phase (27 June to 2 August) likely coincided with the peak in vegetation production and, thus, small mammal abundance (Borchgrevink et al., 2010). Consequently, coyote predation on neonates may be opportunistic during peaks in small mammal abundance (Svoboda et al., 2019).

Coincident with peaks in resource selection, coyotes aligned fine‐scale movement behaviors with areas of high relative probability of use by mule deer and did so to the strongest degree during the birth pulse (Figure 2). Though subtle, coyotes altered searching strategy relative to the location and timing of availability in neonatal prey, which could be consistent with searching that was characterized by slow, complex movement paths during peak parturition (Figure 2). Movement behaviors consistent with searching should occur within patches where such behavior would be most profitable (i.e., increased detection). Coyotes presumably attempted to increase detection of neonatal prey by decreasing movement speed and increasing path complexity (i.e., increased intensity of use), indicating that profitability of neonatal prey was great enough for search behavior to be initiated.

The energetic profitability offered by pulses in availability of neonatal ungulates during peak parturition likely exceeds that of high densities of primary prey (Petroelje et al., 2013). Indeed, in many systems, neonatal deer were the leading diet item of coyotes despite high densities of primary prey during summer months (Patterson et al., 1998). Our observations of selection and fine‐scale movement behavior of coyotes indicate that abundance of primary prey played a negligible role in the shifts in selection and fine‐scale movement behavior during Parturition and that coyotes actively seek neonatal prey as a means to increase energetic gain for a limited time period. Future work that evaluates optimal foraging and its context within the density of primary prey will provide important insights into the effects of primary prey on predator behavior and the cascading effects on alternative prey such as neonatal ungulates (Brunet et al., 2023).

Foraging behavior of pack‐living animals such as coyotes may be influenced by social hierarchy (Gilbert‐Norton et al., 2013). We observed differences in resource selection and movement behaviors not only across reproductive periods but also across social ranks of coyotes (i.e., residents versus transients). We observed inconsistent selection and variable movement patterns relative to mule deer by transient coyotes, ostensibly because of their wide‐ranging movement and ultimately, unstable home ranges (Hinton et al., 2015; Webster et al., 2022; Figure 1). Such findings suggest that predation on neonates by transient coyotes largely is opportunistic, and residents are likely responsible for targeted predation efforts on neonates. Indeed, coyotes were a common predator of neonate mule deer in our study area and most commonly within 2 weeks of birth.

Because coyotes are a widespread predator, prey likely do not have the ability to select home ranges that avoid coyotes, especially if coyotes shift their behavior to where prey exist on the landscape. Thus, microhabitat adjustments and maternal defense by parturient females may be crucial to reduce the risk of predation to neonates. Female ungulates use habitats with increased cover to reduce detection by predators, especially when crypsis is the only form of predator defense for neonates (Barbknecht et al., 2011; Grovenburg et al., 2010; Kjellander et al., 2012; Pitman et al., 2014). Coyotes rely on visual and olfactory cues to locate prey (Pierce et al., 2000; Wells & Bekoff, 1982; Wells & Lehner, 1978); therefore, parturient mule deer may serve as a cue, indicating that neonates are present. Consequently, additional maternal defense tactics may be necessary to prevent predation attempts, especially when maternal defense tactics are minimally risky to the survival of parturient females (Grovenburg et al., 2009; Hamel & Côté, 2009; Lent, 1974; Lingle et al., 2005; Lingle & Pellis, 2002). Indeed, maternal investment plays a crucial role in the survival of neonates against predators. Coyotes have been implicated as a primary predator to neonatal ungulates in countless systems (Ballard et al., 2001; Chitwood et al., 2015; Kilgo et al., 2014; Linnell et al., 1995; Shuman et al., 2017), but consideration of the behavioral mechanisms by which coyotes are effective predators in light of prey defense tactics has remained less understood.

Our results are based on models of the relative probability of use by mule deer that incorporate many spatial covariates (e.g., shrub cover, and slope) that are most likely to influence the probability of use by mule deer. These models represent areas on the landscape wherein mule deer are likely to occur at each reproductive period. Although seemingly correlative, the temporal shifts in resource selection and movement by coyotes are indicative of selection for mule deer habitat. Furthermore, the use of habitat features (i.e., proxies) such as shrub cover may not be applicable when habitat features do not align with the spatial distribution of prey. Therefore, evaluating the responses of coyotes solely through habitat attributes instead of the lens of prey could have different interpretations for coyote behavior. We modeled the relative probability of use by mule deer across the landscape with data that incorporated habitat features to produce a single composite layer that reflects spatial variation in the relative probability of use by mule deer across reproductive periods. Therefore, our results should be directly comparable to the spatial and temporal variation in mule deer presence and provide the necessary context to understand coyote behavior relative to the spatial and temporal distribution of neonatal prey.

Coyotes should invest time foraging and exhibit search behavior in areas where prey are most likely to occur (Chamberlain et al., 2021; Fauchald & Tveraa, 2003). Indeed, resident coyotes selected areas of high relative probability of use by mule deer and did so during peak parturition when neonates were most abundant and vulnerable, whereas transients exhibited inconsistent selection across reproductive periods (Figure 1). Furthermore, resident coyotes engaged in search behavior during peak parturition, whereas movement behavior by transients was much less predictable (Figure 2). The behavior exhibited by coyotes yields additional insight into the mechanisms by which generalist predators can affect the survival of ungulate prey, and when those effects may manifest. Our analyses reveal a link between foraging theory and predator ecology to demonstrate the behavioral mechanisms underlying predation by a generalist predator and potential effects on the survival of neonatal ungulates.

## AUTHOR CONTRIBUTIONS

**Katey Huggler:** Conceptualization (equal); data curation (lead); formal analysis (lead); funding acquisition (supporting); writing – original draft (lead); writing – review and editing (lead). **Matthew M Hayes:** Data curation (equal); writing – review and editing (supporting). **Patrick W. Burke:** Data curation (equal); writing – review and editing (supporting). **Mark Zornes:** Data curation (equal); writing – review and editing (supporting). **Daniel Thompson:** Data curation (equal); writing – review and editing (supporting). **Patrick Lionberger:** Data curation (equal); writing – review and editing (supporting). **Miguel Valdez:** Data curation (equal); writing – review and editing (supporting). **Kevin L. Monteith:** Conceptualization (equal); data curation (lead); funding acquisition (lead); writing – review and editing (equal).

## CONFLICT OF INTEREST STATEMENT

The authors declare no conflicts of interest.

## Data Availability

Data used for analyses are stored in Dryad Digital Repository; DOI: 10.5061/dryad.fbg79cp1f.

## APPENDIX A

**TABLE A1:** Sample sizes and number of GPS relocations used in RF models of the probability of use by female deer, and models of resource selection and movement behavior of coyotes in southwest Wyoming, USA, from 2018 to 2019.

Species	Pre‐parturition		Parturition		Hider		Follower	
	Locations	*N*	Locations	*N*	Locations	*N*	Locations	*N*
Coyote	10,349	27	31,010	27	29,784	27	29,662	27
Mule deer	46,130	56	40,412	56	33,060	7656	27,027	56
